# Supplementary material for: Causal interplay between lactose intolerance and gut microbiota: a combined bidirectional Mendelian randomization and in vivo validation study
Source: Front Nutr. 2026 Jun 1;13:1803337. doi: 10.3389/fnut.2026.1803337 (PMC13265576; doi:10.3389/fnut.2026.1803337)
Supplement: Supplementary file 1 [file Data_Sheet_1.zip › supplementary materials/Forward/leave one out cross validation/ebi-a-GCST90027778.finngen_R12_E4_LACTONAS.pdf]

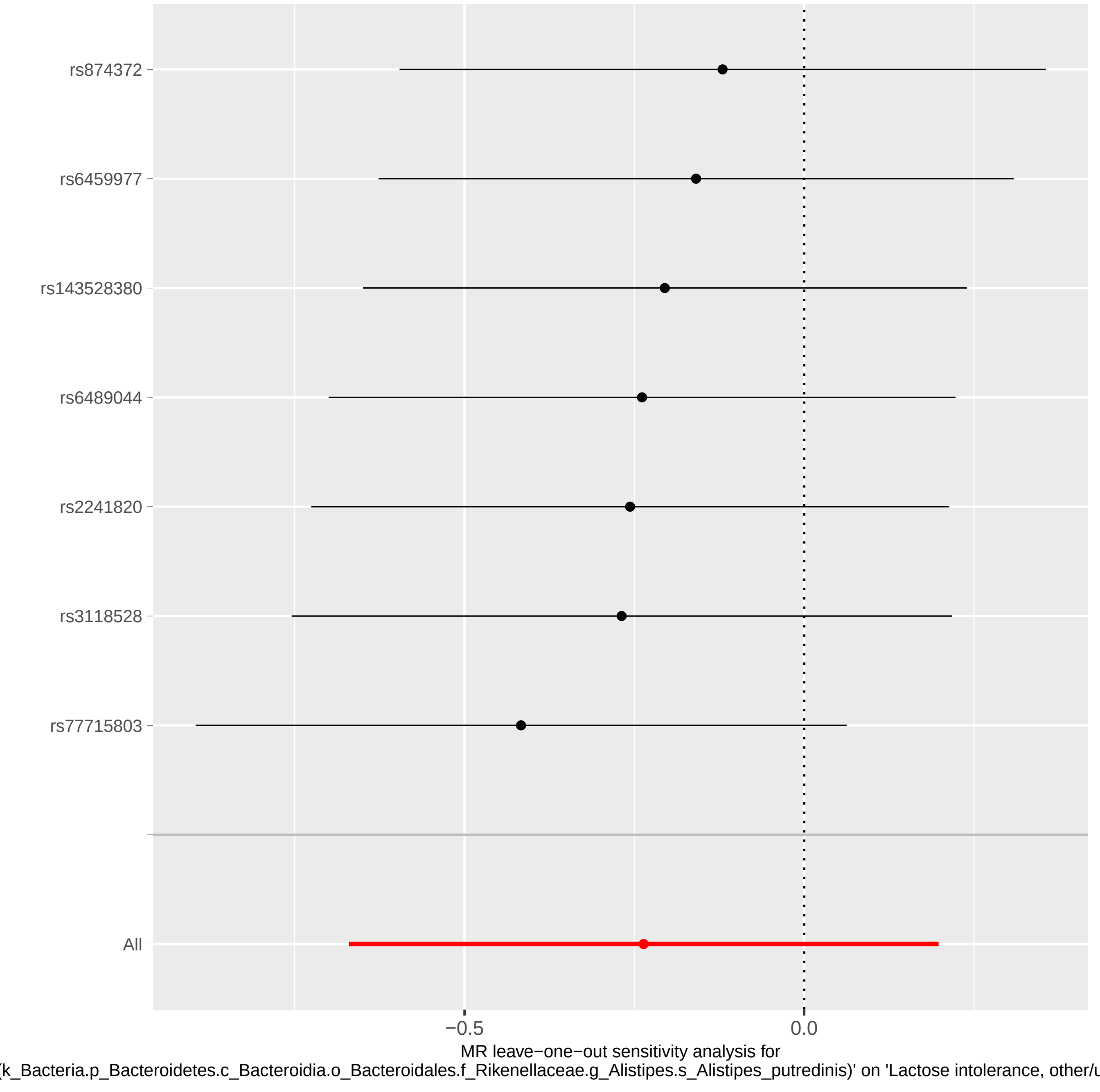

rs874372
rs6459977
rs143528380
rs6489044
rs2241820
rs3118528
rs77715803
All
−0.5
0.0
MR leave−one−out sensitivity analysis for
(k_Bacteria.p_Bacteroidetes.c_Bacteroidia.o_Bacteroidales.f_Rikenellaceae.g_Alistipes.s_Alistipes_putredinis)' on 'Lactose intolerance, other/u
